# Race, Intergenerational Social Mobility and Stressful Life Events

**DOI:** 10.3390/bs8100086

**Published:** 2018-09-20

**Authors:** Shervin Assari

**Affiliations:** 1 Department of Psychology, University of California, Los Angeles (UCLA), Los Angeles, CA 90095, USA; assarish@ucla.edu or assari@umich.edu; Tel.: +1-310-206-5162; 2 BRITE Center for Science, Research and Policy, University of California, Los Angeles (UCLA), Los Angeles, CA 90095, USA; 3 Center for Research on Ethnicity, Culture, and Health (CRECH), School of Public Health, University of Michigan, Ann Arbor, MI 48104, USA; 4 Department of Psychiatry, University of Michigan, 4250 Plymouth Rd., Ann Arbor, MI 48109-2700, USA

**Keywords:** African Americans, socioeconomic status (SES), social mobility, racism, perceived stress

## Abstract

*Background.* Socioeconomic status (SES) has smaller protective effects on the health of African Americans, and the differential association between social mobility and stress may explain the diminished returns of SES for African Americans. *Aim.* This study tested the race/ethnic differences in the association between upward and downward social mobility and stress in a nationally representative sample of African American and White American adults. *Methods.* This study included 3570 African Americans and 891 non-Hispanic *White Americans* from the National Survey of American Life (NSAL), 2003. Race/ethnicity, gender, age, upward and downward social mobility (independent variable, defined as difference between parent and respondent education), and stressful life events (SLE, dependent variable) were measured. Linear regression models were used for data analysis. *Results.* In the pooled sample that included both races, upward and downward social mobility were both associated with SLE, the net of all covariates. Significant interactions were found between race/ethnicity and social mobility, suggesting a stronger association between social mobility and stress for *White Americans* than for African Americans. According to race-stratified models, upward and downward social mobility were associated with higher SLE for *White Americans* but not *African Americans*. *Conclusion.* Although upwardly and downwardly mobile *White Americans* experience more stress than the socially stable *White Americans*, African Americans do not experience a change in SLE related to their intergenerational social mobility.

## 1. Introduction

Socioeconomic status (SES) is a fundamental factor that operates as a social determinant of health [1,2,3,4,5,6,7,8,9], and a major cause of health disparities by race/ethnicity [10]. Although much is known about the overall protective effects of SES [1,2,3], less is known about how racial and ethnic variations in high SES translate to health [11,12,13,14]. For example, while several longitudinal studies have shown that populations with the highest SES report the best health [4,5,6,7,8,9,15], new analyses of national studies [16,17,18,19] suggest that the protective effects of high SES [20,21,22] are systemically smaller for African Americans [23,24] and other racial/ethnic minority populations [25], compared to *White Americans*. The weaker effect of SES observed in African Americans compared to *White Americans* is documented for the effects of educational attainment on alcohol drinking behaviors [26], smoking [27], diet [28], sleep [16], suicide [29] body mass index (BMI) [30], and life expectancy [26]. Additionally, the effects of income on mental health [13], chronic disease [31], obesity [32], and oral health [33] are all smaller for African Americans than *White Americans*. These patterns occur in children [32], youth [19], adults [11,13], and older adults [16,27], and they persist for other SES indicators such as employment [34] and marital status [14,30]. These patterns are also observed in cross-sectional [29] as well as longitudinal data [17]. The same patterns are shown within individuals [27] as well as trans-generationally [14,30].

Not only does SES generate smaller health gains, high SES African Americans report worse mental health compared to low SES African Americans [17,18,19,29,35,36,37]. Although this pattern is more evident for high SES African American males, it has been shown across age groups [17,19,35]. However, it remains unclear why the risk of depression [12,38] and depressive symptoms [17,39] are higher in high SES African Americans (particularly males) than their lower SES counterparts. To provide some examples, in the Americans’ Changing Lives (ACL) study, higher educated African American men reported an increase in depressive symptoms over the 25 years of follow up [17]. In the 2003 National Survey of American Life (NSAL) adult sample, higher education increased the risk of suicidal ideation among Caribbean Black females [29]. In addition, the NSAL data showed that higher household income increased the risk of depression for African American males [12].

There is an economic explanation for the diminished returns experienced by African Americans. Upward social mobility (i.e., change in social status based on education, wealth, and occupation) and SES resources such as education generates far less income for African Americans compared to *White Americans* [40,41]. Labor market preferences and practices [42], residential segregation [43], differential quality of education [40,41], and interpersonal discrimination [38,39,44] may all play a role. Some recent research suggests that various types of stress such as racial discrimination may have a role in explaining the diminished returns of SES for African Americans [38]. For instance, high SES African Americans continue to report high levels of race-related stress. However, high SES reduces stress for *White Americans* [39,44]. Race-related stress is prevalent among high SES African Americans because they likely have more contact with *White Americans* [39,44], in their neighborhoods and workplaces. Still, the exact role of various types of stress on the unequal health gains experienced by *White Americans* and African Americans has yet to be fully understood.

### Aims

This study investigates racial differences in the association between intergenerational social mobility (i.e., group changes in status relative to their parents) and stressful life events (SLE). Utilizing a representative sample of American adults, we expected that upwardly mobile African Americans will still report high SLE, suggesting that upward mobility does not reduce SLE for African Americans, as it does for *White Americans*. In statistical terms, it is hypothesized that the relationship between social mobility on SLE will be moderated by race/ethnicity.

The results of our investigation will help us understand why high SES is more protective for *White Americans* than African Americans [31,32,40,41], and why high SES operates as a vulnerability factor for some groups of African Americans [38], as explained by the diminished returns theory [23,24]. This theory posits that SES generates smaller tangible outcomes for racial and ethnic minorities, compared to Whites [23,24]. Given that race impacts how society treats individuals, African Americans who have successfully climbed the social ladder still face blocked opportunities and racism [45,46]. The results will suggest whether social mobility has universal or different implications for the health and well-being of African Americans and *White Americans*. As stressful life events (SLE) [47] and other stress [19,45,46,48,49,50] are a strong determinant of health, differential exposure to SLE may be partially responsible for racial/ethnic differences in health across each social status [50]. 

## 2. Methods

### 2.1. Design

The National Survey of American Life is a cross-sectional mental health survey of non-Hispanic White and non-Hispanic Black adults in the US [51,52,53]. Although NSAL methods and sampling are well-explained elsewhere [51,52,53], we will briefly report the study here. 

### 2.2. Participants and Sampling

The analytical sample of this study included a total number of 4461 individuals who were either non-Hispanic White (*n* = 891) or African American (*n* = 3570). The NSAL applied a household probability sampling to draw a national sample. The NSAL African Americans and non-Hispanic *White Americans* were selected from rural areas, large cities, and other urban areas [51,52,53]. With a multi-stage sampling design, the NSAL used a core national sample of African Americans and *White Americans*. The NSAL participants were adults (aged 18 years and older) who resided in the coterminous US (48 states). Participants were restricted to non-institutionalized individuals who could conduct a structured interview in English. Thus, individuals were excluded if they were in nursing homes, long-term medical care settings, prisons, and jails or were non-English speakers [51,52,53]. 

### 2.3. Data Collection

Structured interviews were used to collect data. Interviews were conducted in English and were performed by race-matched interviewers. About 82% of total interviews were face-to-face; while the 14% remaining were conducted as telephone interviews. NSAL used Computer-Assisted Personal Interviewing (CAPI) for all the face-to-face interviews. CAPI uses computers to assist the process of answering lengthy questionnaires with multiple skip patterns. CAPI enhances data quality for long and complex surveys [54]. Interviews took about 140 minutes to complete. The response rate was 71% for African Americans and 70% for non-Hispanic *White Americans*.

### 2.4. Measures

The variables employed in this study were race/ethnicity, age, household income, education attainment, and perceived (every day) discrimination.

*Race/Ethnicity.* NSAL measured race/ethnicity as self-identified race and ethnicity. Participants self-identified either as non-Hispanic African Americans or non-Hispanic *White Americans*. Non-Hispanic African Americans were defined as Black without any ancestral ties to the Caribbean countries.

*Education Attainment.* Years of education were measured using self-reporting. This variable was treated as a continuous measure ranging from 4 to 17. 

*Intergenerational Mobility.* Upward and downward intergenerational social mobility was defined based on a comparison between a respondent’s level of education and that of their parent(s). Participants were asked about their own and their parents’ highest year of education. Separate questions were asked for the number of years of schooling their mother (or the woman who raised them) and father (or the man who raised them). If education information was available only for one parent, that parent’s level of education was used. For respondents who reported education information for both parents, we used the parent with the highest level of education. Intergenerational mobility included upward social (individual has higher education than their parents), no social mobility (equal education of respondent and parents), and downward social mobility (respondent has a lower education than their parents). The reference group was no social mobility [55].

### 2.5. Statistical Analysis

To accommodate the NSAL sampling weights, Stata 13.0 (Stata Corp.; College Station, TX, USA) was used for data analysis. Taylor series linearization was applied for the re-estimation of design-based standard errors (SEs). As our analysis was limited to *White Americans* and African Americans, we used sub-population survey commands.

To describe the sample, we used survey mean and proportions (%), which are reported for the pooled sample as well as by race/ethnicity. To compare *White Americans* and African Americans for study variables, we applied independent sample and Chi-square tests. For multivariable analysis, we used linear regression models. From the regression models, the adjusted beta (b), 95% confidence intervals (CIs), z, and *p* levels are reported. 

We ran four linear regression models overall. In all of them, either downward or upward social mobility was the independent variable, SLE was the dependent variable, and age, gender, education, and marital status were covariates. First, linear regression models were estimated in the pooled sample of *White Americans* and African Americans, in the absence and presence of interaction terms. Then additional models were conducted for each racial/ethnic group. Model 1 did not include the race/ethnicity by social mobility interaction terms; however, Model 2 included the race/ethnicity by social mobility interaction terms. Model 3 and Model 4 were estimated for *White Americans* and African Americans, respectively.

## 3. Results

### 3.1. Descriptive Statistics

A total number of 4461 individuals entered the study who were either non-Hispanic White (*n* = 891) or African American (*n* = 3570). Table 1 shows the descriptive statistics in the pooled sample and also by race/ethnicity. Statistically significant differences were detected between *White Americans* and African Americans regarding years of education, household income, SLE, and intergenerational social mobility (*p* < 0.05). *White Americans* reported higher years of education and household income in comparison to African Americans. *White Americans* reported lower SLE than African Americans.

### 3.2. Linear Regressions for Upward Social Mobility

Table 2 shows the summary of the results of four nested linear regression models with upward social mobility as the predictor and SLE as the outcome. The first two models were in the pooled sample. The other two linear regression models were for *White Americans* and for African Americans. Model 1, which only included main effects, showed a significant positive association between upward social mobility and SLE, the net of all covariates. Model 2 showed a significant interaction between race/ethnicity and upward social mobility, showing a smaller association between upward social mobility and SLE for African Americans than *White Americans*. 

### 3.3. Linear Regressions for Downward Social Mobility

Table 3 shows the summary of the results of four nested linear regression models with downward social mobility as the predictor and SLE as the outcome. The first two models were in the pooled sample. The other two linear regression models were for *White Americans* and for African Americans. Model 1, which only included the main effects, showed a significant positive association between downward social mobility and SLE, the net of all covariates. Model 2 showed a significant interaction between race/ethnicity and downward social mobility, showing a smaller association between downward social mobility and SLE for African Americans than *White Americans*. 

## 4. Discussion

Using a nationally representative sample of American adults, intergenerational social mobility was associated with SLE. However, this association was significant for non-Hispanic *White Americans* but not African Americans. Specifically, while non-Hispanic *White Americans*’ SLE was a function of intergenerational social mobility, African Americans’ SLE was constantly high regardless of intergenerational social mobility status.

The results show that African Americans experience high SLE, regardless of their social mobility, which is a different pattern compared to *White Americans* whose SLE is a function of social mobility. These results are in support of the minorities’ diminished returns theory, which argues that increases in SES generate less health for minorities [31,32,40,41] and high SES may operate as a vulnerability factor for African Americans’ mental health [38].

The results contribute to a growing literature on the intersection of race/ethnicity, class, stress, and mental health. Some research has documented worse mental health among high SES African Americans, particularly for males [17,18,19,35,36,37]. In a recent study using the 2017 State of the State Survey (SOSS) data, income was found to protect self-rated mental health for *White Americans* but not African Americans [13].

Stress is one of the root causes of the health inequities observed between African Americans and *White Americans*. Supporting this argument, a longitudinal study with 18 years of follow-up found that African American youth from high income families who resided in predominantly White areas reported higher race-related stress between ages 9 to 37 years [56]. African American youth from high SES families who resided in predominantly White areas were also found to be more depressed—an association which was fully explained by race-related stress [39]. 

The major contribution of this study is that it shows a differential link between upward social mobility and SLE by race. Other types of stress such as race-based discrimination (perceived discrimination), and goal striving stress (GSS) may explain why economic resources have smaller health effects for African Americans than *White Americans* [31,32,40,41]. Stress may explain some of the Black–White differences in physical and mental health, while providing insight into why high SES African Americans may be more vulnerable and sensitive to stress than lower SES African Americans [44,57]. In a study of African American adolescents, high SES individuals were more vulnerable to the effects of race-related stress on the risk of depression [57]. In addition to SES, male gender may also increase the sensitivity of African Americans to race-related stress [58,59,60]. 

The result questions whether the US is the land of opportunity for all racial and ethnic groups. In contrast to non-Hispanic *White Americans*, whose intergenerational social mobility impacts their SLE, all African Americans continue to experience high SLE regardless of their social mobility. We argue that for African Americans, SLE is mainly a function of race, not class, which is very different from the pattern for non-Hispanic *White Americans*, for whom SLE is a function of class. This finding is consistent with other research that suggests that class-based stress [61] may differently impact the health of *White Americans* and African Americans.

Our findings have implications for how the stress associated with social mobility is related to the mental health of *White Americans* and African Americans. Hudson et al. [36,37,62], Fuller-Rowell et al. [63] and Steele [64] have shown that upward social mobility brings less health benefits for African Americans than *White Americans*. High discrimination may explain African Americans’ diminished health gains from SES [65]. John Henryism, a psychologically and physiologically taxing coping style that is commonly used by African Americans for upward social mobility, is suggested to explain why high SES adds to the psychological costs for this group [18,50,66]. Our results propose stress and SLE as another possible mechanism for this phenomenon. The psychosocial gains and losses that accompany social mobility are a function of how difficult upward mobility is given one’s race/ethnicity, gender, and environment. We know that upward social mobility has different effects on the health of population subgroups [63]. We suggest that SES mobility effects may operate through SLE and other stressors differently for *White Americans* and African Americans. 

### 4.1. Limitations

Our study has several limitations. First, because of its cross-sectional design, our results should not to be interpreted as causal. Future research should study these patterns over time using longitudinal data. While upward and downward social mobility impact stress, stress may also impact social mobility. As the current study only controlled for a few confounders, future research should include a more inclusive list of covariates such as childhood SES, area level SES, occupation, and racial composition at work and in the neighborhood. There is a specific need to explore these associations for males and females separately. Despite the above methodological limitations, the results extend the existing literature as only a handful of studies have studied racial variation in the stress associated with social mobility [19,45,46,48,49,50]. 

### 4.2. Future Research

More research should be done on the intersections of race and class in shaping social patterns of stress and resilience, which ultimately shape the health of African Americans and *White Americans*. To date, it remains unclear how stress is shaped by the intersection of race, place, gender, and class. It is also unknown how African Americans show a relative advantage compared to *White Americans* in dealing with SLE [67]. It is not clear whether previous experiences of stress during life prepares them for future exposure to stress. Future research should also define downward and upward social mobility by comparing parents’ and individual’s occupations, and such a study could explain some of the reasons that there are differential correlates of social mobility by race. Finally, more research is needed using an intersectionality framework, and research should also include contextual and ecological factors such as neighborhood stress and SES that may differently impact the distribution of stress for *White Americans* and African Americans. 

## 5. Conclusions

To summarize, our research findings suggest that upward and downward social mobility are not similarly related to SLE for African Americans and *White Americans*. The socioeconomic correlates of SLE are not universal but conditional upon on race/ethnicity. How race/ethnicity, class, and SLE affect populations is not linear and additive but non-linear and multiplicative [17,18,19,35,36,37], with social mobility associated with change in SLE for *White Americans* but not African Americans. These findings are consistent with the theory of minorities’ diminished returns [23,24], which suggests that SES is less consequential for non-White than *White Americans*. African Americans experience SLE, regardless of their social mobility, a pattern that is very different for *White Americans*.

## Figures and Tables

**Table 1:** Descriptive statistics in the pooled sample and by race/ethnicity.

	All (*n* = 4461)		African American (*n* = 891)		Non-Hispanic White (*n* = 891)	
	% (SE)	95% CI	% (SE)	95% CI	% (SE)	95% CI
Gender						
Male	45.69 (0.01)	43.43–47.97	44.03 (0.01)	42.35–45.72	47.26 (0.02)	42.89–51.66
Female	54.31 (0.01)	52.03–56.57	55.97 (0.01)	54.28–57.65	52.74 (0.02)	48.34–57.11
Social Mobility *						
Downward	22.61 (0.02)	19.24–26.37	20.79 (0.01)	19.02–22.67	24.32 (0.03)	18.05–31.92
None	24.71 (0.01)	22.36–27.23	22.47 (0.01)	20.81–24.21	26.83 (0.02)	22.41–31.76
Upward	52.68 (0.02)	49.50–55.85	56.75 (0.01)	54.35–59.11	48.85 (0.03)	43.34–54.39
	**Mean (SE)**	**95% CI**	**Mean(SE)**	**95% CI**	**Mean(SE)**	**95% CI**
Age *	43.54 (0.71)	42.11–44.97	42.07 (0.53)	40.98–43.16	44.90 (1.31)	42.10–47.70
Education *	12.92 (0.16)	12.60–13.25	12.46 (0.08)	12.29–12.63	13.35 (0.29)	12.73–13.97
SLE *	0.65 (0.02)	0.61–0.69	0.79 (0.02)	0.74–0.84	0.52 (0.02)	0.48–0.57

Notes: Standard Error (SE), Confidence Interval (CI), Stressful Live Events (SLE); * *p* < 0.05.

**Table 2:** Linear regressions between upward social mobility and stress.

	b (SE)	95% CI	t	*p*
***Model 1***				
Upwardly mobile	0.06 (0.03)	−0.01–0.12	1.74	0.088
Race (African Americans)	−0.23 (0.04)	−0.30–0.15	−6.14	**0.000**
Gender (Female)	0.11 (0.05)	0.01–0.20	2.23	**0.031**
Age	−0.01 (0.00)	−0.01–0.01	−5.82	**0.000**
Education (Years)	−0.03 (0.01)	−0.05–0.02	−4.20	**0.000**
Intercept	3.74 (0.41)	2.92–4.55	9.21	**0.000**
***Model 2 (Model 1 + Interactions)***				
Upwardly mobile	0.12 (0.05)	0.02–0.21	2.42	**0.019**
Race (African Americans)	0.32 (0.05)	0.22–0.42	6.18	**0.000**
Gender (Female)	0.11 (0.05)	0.01–0.20	2.28	**0.027**
Age	−0.01 (0.00)	−0.01–0.01	−5.64	**0.000**
Education (Years)	−0.03 (0.01)	−0.04–0.02	−4.17	**0.000**
Race * Upwardly mobile	−0.13 (0.06)	−0.26–0.01	−2.10	**0.041**
Intercept	1.17 (0.10)	0.96–1.37	11.22	**0.000**
***Model 3 (White Americans)***				
Upwardly mobile	0.00 (0.04)	−0.08–0.08	0.02	0.983
Gender (Female)	0.11 (0.03)	0.05–0.18	3.51	**0.001**
Age	−0.01 (0.00)	−0.01–0.01	−10.17	**0.000**
Education (Years)	−0.04 (0.01)	−0.05–0.03	−8.26	**0.000**
Intercept	1.66 (0.10)	1.47–1.86	17.39	**0.000**
***Model 4 (African Americans)***				
Upwardly mobile	0.11 (0.05)	0.00–0.21	2.15	**0.049**
Gender (Female)	0.11 (0.08)	−0.07–0.28	1.25	0.229
Age	−0.01 (0.00)	−0.01–0.00	−2.96	**0.010**
Education (Years)	−0.02 (0.01)	−0.05–0.01	−1.59	0.133
Intercept	0.99 (0.16)	0.66–1.33	6.32	**0.000**

Outcome: Stressful Life Events (SLE); bold numbers are significant.

**Table 3:** Linear regressions between downward social mobility and stress.

	b (SE)	95% CI	t	*p*
***Model 1***				
Downwardly mobile	0.11 (0.05)	0.02–0.20	2.42	**0.019**
Race (African Americans)	−0.22 (0.04)	−0.31–0.13	−5.02	**0.000**
Gender (Female)	0.14 (0.03)	0.07–0.20	4.16	**0.000**
Age	−0.01 (0.00)	−0.01–0.00	−5.68	**0.000**
Education (Years)	−0.05 (0.01)	−0.06–0.04	−8.12	**0.000**
Intercept	3.88 (0.43)	3.01–4.75	8.98	**0.000**
***Model 2 (Model 1 + Interactions)***				
Downwardly mobile	0.18 (0.07)	0.04–0.32	2.50	**0.016**
Race (African Americans)	0.30 (0.05)	0.20–0.40	5.99	**0.000**
Gender (Female)	0.14 (0.03)	0.07–0.20	4.25	**0.000**
Age	−0.01 (0.00)	−0.01–0.00	−5.59	**0.000**
Education (Years)	−0.05 (0.01)	−0.06–0.04	−8.58	**0.000**
Race * Upwardly mobile	−0.15 (0.09)	−0.33–0.02	−1.80	0.078
Intercept	1.37 (0.12)	1.14–1.60	11.90	**0.000**
***Model 3 (White Americans)***				
Downwardly mobile	0.03 (0.04)	−0.06–0.12	0.70	0.489
Gender (Female)	0.16 (0.05)	0.06–0.27	3.13	**0.004**
Age	−0.01 (0.00)	−0.01–0.00	−5.60	**0.000**
Education (Years)	−0.05 (0.01)	−0.07–0.03	−4.72	**0.000**
Intercept	1.59 (0.16)	1.27–1.91	10.10	**0.000**
***Model 4 (African Americans)***				
Downwardly mobile	0.17 (0.07)	0.03–0.32	2.49	**0.025**
Gender (Female)	0.12 (0.04)	0.03–0.20	2.93	**0.010**
Age	−0.01 (0.00)	−0.01–0.00	−3.81	**0.002**
Education (Years)	−0.05 (0.01)	−0.07–0.04	−7.21	**0.000**
Intercept	1.42 (0.15)	1.10–1.75	9.29	**0.000**

Outcome: Stressful Life Events (SLE); bold numbers are significant.
